# Extrinsic Calibration between Camera and LiDAR Sensors by Matching Multiple 3D Planes †

**DOI:** 10.3390/s20010052

**Published:** 2019-12-20

**Authors:** Eung-su Kim, Soon-Yong Park

**Affiliations:** 1 School of Computer Science & Engineering, Kyungpook National University, Daegu 41566, Korea; jsm80607@naver.com; 2 School of Electronics Engineering, Kyungpook National University, Daegu 41566, Korea

**Keywords:** camera, LiDAR, calibration, plane matching, ICP, projection

## Abstract

This paper proposes a simple extrinsic calibration method for a multi-sensor system which consists of six image cameras and a 16-channel 3D LiDAR sensor using a planar chessboard. The six cameras are mounted on a specially designed hexagonal plate to capture omnidirectional images and the LiDAR sensor is mounted on the top of the plates to capture 3D points in 360 degrees. Considering each camera–LiDAR combination as an independent multi-sensor unit, the rotation and translation between the two sensor coordinates are calibrated. The 2D chessboard corners in the camera image are reprojected into 3D space to fit to a 3D plane with respect to the camera coordinate system. The corresponding 3D point data that scan the chessboard are used to fit to another 3D plane with respect to the LiDAR coordinate system. The rotation matrix is calculated by aligning normal vectors of the corresponding planes. In addition, an arbitrary point on the 3D camera plane is projected to a 3D point on the LiDAR plane, and the distance between the two points are iteratively minimized to estimate the translation matrix. At least three or more planes are used to find accurate external parameters between the coordinate systems. Finally, the estimated transformation is refined using the distance between all chessboard 3D points and the LiDAR plane. In the experiments, quantitative error analysis is done using a simulation tool and real test sequences are also used for calibration consistency analysis.

## 1. Introduction

Three-dimensional mapping and localization techniques for autonomous vehicles [1,2,3] have been investigated extensively for over the years. In recent investigations, visual and depth information is commonly used to reconstruct accurate 3D maps containing shape and color information of an environment. Especially, a 360-degree LiDAR sensor and multi-view vision cameras are commonly used to acquire omnidirectional shape and color information from a vehicle. Accurate extrinsic calibration between cameras and LiDAR sensors increase the reliability of data fusions between the color and shape information of 3D point data.

Many extrinsic calibration methods between 360-degree LiDAR and vision camera have been proposed. However, they mostly require a specially designed calibration board or calibration object [4,5,6,7,8]. Zhou et al. [4] used a planar calibration board that has three circular holes. The center coordinates of the circular holes with respect to both the LiDAR and camera are used to align the two sensor’s coordinate axes. Pusztai and Hajder [5] proposed an extrinsic calibration method using a cube-type object. The 3D faces of the cube are fitted to planes using 3D points from a 3D LiDAR and they are again used to find the 3D corners of the cube. The relative rotation between the camera–LiDAR system is estimated by solving 2D and 3D correspondences of the cube corners. Lee et al. [6] use a spherical object for extrinsic calibration. First, the 3D center coordinates of the spherical object are estimated using the 3D LiDAR point scanning the surface of the object. The 2D center coordinates of the spherical object are found by a circle detection algorithm in the camera images. Using the 2D and 3D correspondences of the center coordinates, the extrinsic parameters are estimated using the PnP algorithm. Their method is simple and provides stable performance. Mirzaei F. M. et al. propose [9] a method for joint estimation of both the intrinsic parameters of the Lidar and the extrinsic parameter between a LIDAR and a camera. This method is based on existing approaches to solving this nonlinear estimation problem. In these cases, the accuracy of the result depends on a precise initial estimate. This method computes an initial estimate for the intrinsic and extrinsic parameters in two steps. Then, the accuracy of the initial estimates is increased by iteratively minimizing a batch nonlinear least-squares cost function.

Instead of using any special calibration board or object, the common chessboard pattern is also used for extrinsic parameter estimation. Pandey et al. [10] proposed a calibration method of using the normal vector of a chessboard. The rotation and translation between a LiDAR and camera are estimated by minimizing an energy function of correspondences between depth and image frames. Sui and Wang [11] proposed to use the 2D edge lines and the 3D normal vector of the chessboard. The boundaries of the chessboard in 2D images are extracted and their 3D line equations are also calculated by the RANSAC of 3D LiDAR data. A single pose of the checkerboard provides one plane correspondence and two boundary line correspondences. Extrinsic parameters between a camera and LiDAR are estimated using these correspondences obtained from a single pose of the chessboard. Huang and Barth [12] propose an extrinsic calibration method using geometric constraints of the views of chessboard from the LiDAR data and camera image. First, the image and distance data at different position and orientation of the chessboard are acquired using a LiDAR and camera system. The rotation and translation are estimated by solving the closed form equation using the normal vector of the chessboard plane and the vector passing along the plane. Finally, these are refined using the maximum likelihood estimation. Velas et al. [13] propose an extrinsic calibration method using the detection of a calibration board containing four circular holes. The coarse calibration of this method is performed using circle centers and the radius of the 3D LiDAR data and 2D image. Then, a fine calibration is performed using the 3D LiDAR data captured along the circle edges and the inverse distance transform of the camera image. Geiger et al. [14] calibrate a stereo camera and a LiDAR in a single shot using multiple chessboards placed across a room. This paper’s algorithm requires that all the chessboards’ corners’ coordinates should be calculated with respect to the stereo camera to distinguish multiple chessboards. To do this, the authors use the stereo camera to reconstruct the 3D coordinates of every chessboard corner. The rotation between the camera and the LiDAR is estimated using the normal vectors between the corresponding 3D LiDAR points and chessboard corners from the camera. The translation is estimated using the point-to-plane distance of 3D centers of the chessboard. Pandey et al. [15] propose a method for extrinsic calibration of a LiDAR and camera system using the maximization of mutual information between surface intensities. Budge et al. [16,17] propose a TOF flash LiDAR and camera method using the chessboard pattern. Their method combines systematic error correction of the flash LiDAR data, correction for lens distortion of the digital camera and flash LiDAR images, and fusion of the LiDAR to the camera data in a single process.

In this paper, we propose an external calibration method that is based on the theory proposed in the work of [10]. However, instead of using a single energy function to calibrate extrinsic parameters, we divide the calibration process into three different steps to increase the reliability of the calibration. In our previous paper [18], we have introduced a two-step calibration method and applied the method to reconstruct a 3D road map for an autonomous vehicle. Rotation and translation calibration steps are done independently to get an initial transformation. Then, the initial results are optimized iterative in the third step of the calibration. More details of the proposed method and the differences from those in the literature are shown in the next sections.

The contents of this paper are as follows. Section 2 briefly describes a multi-sensor device used for calibration experiments. Section 3 is divided into three subsections. Section 3.1 explains a plane fitting method of the chessboard using the LiDAR sensor data. Section 3.2 explains another plane fitting method of the chessboard with respect to the camera coordinate system using the reprojected 2D chessboard corners. Rotation and translation estimation between a camera–LiDAR unit is described in Section 3.3. Refinement of the initial rotation and translation for more accurate calibration is described in Section 3.4. Section 4.1 and Section 4.2 show the experimental results and performance analysis using simulation and real test sequences, respectively. Finally, conclusions are provided in Section 5.

## 2. An Omnidirectional Camera–LiDAR System

**Figure 1:**
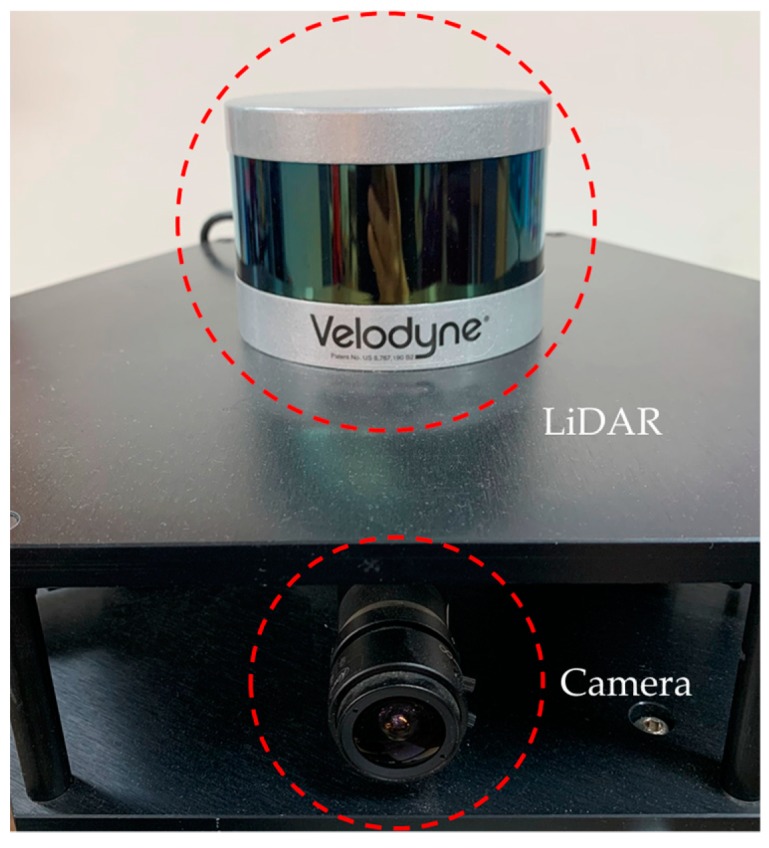
An image of our camera–LiDAR imaging system.

A front view of our camera–LiDAR system is shown in Figure 1. The system is originally designed to obtain 360 degree images using multiple cameras and LiDAR data for autonomous navigation of a vehicle. The system can be mounted on top of the vehicle and obtain the color and shape data around the vehicle in real-time. To align the color and shape information from the two different types of sensors, it needs to calibrate the extrinsic parameters between sensors. In this paper, however, we propose a calibration algorithm that finds the external parameters between a single camera and the LiDAR. The proposed method can later be used to calibrate multiple cameras with the LiDAR by sequentially running the proposed algorithm to each camera–LiDAR pair.

**Figure 2:**
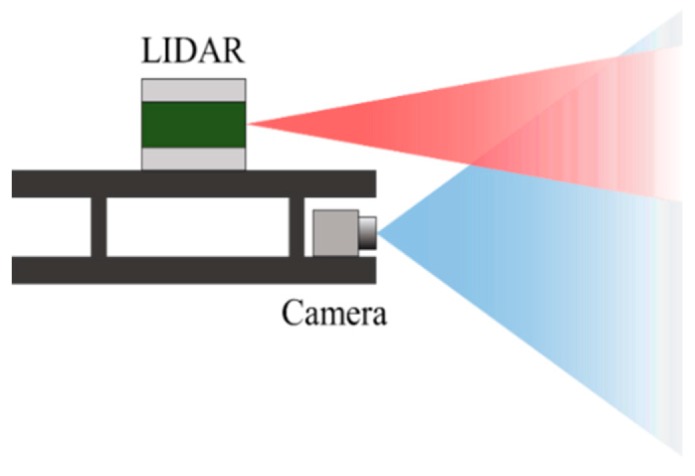
A diagram of a camera and LiDAR unit on a plate. The fields of view of the camera (blue) and the LiDAR (red) partly overlap each other.

For color image acquisition, a FLIR Blackfly color camera is used. The field of view of the camera lens is 103.6°, and the resolution is 1280 × 1024 pixels. The camera is mounted on the top of a hexagonal plate. In addition, a 16-channel Velodyne VLP-16 LiDAR sensor is mounted on another plate and fixed above the six cameras. The VLP-16 sensor uses an array of 16 infrared (IR) lasers paired with IR detectors to measure distances to objects. The vertical field of view of the sensor is 30°, and it can acquire distance data at a rate of up to 20 Hz for 360 degree horizontally. Our camera–LiDAR system has a partially overlapping field of view, as shown in Figure 2.

## 3. Extrinsic Calibration of the Camera–LiDAR System

**Figure 3:**
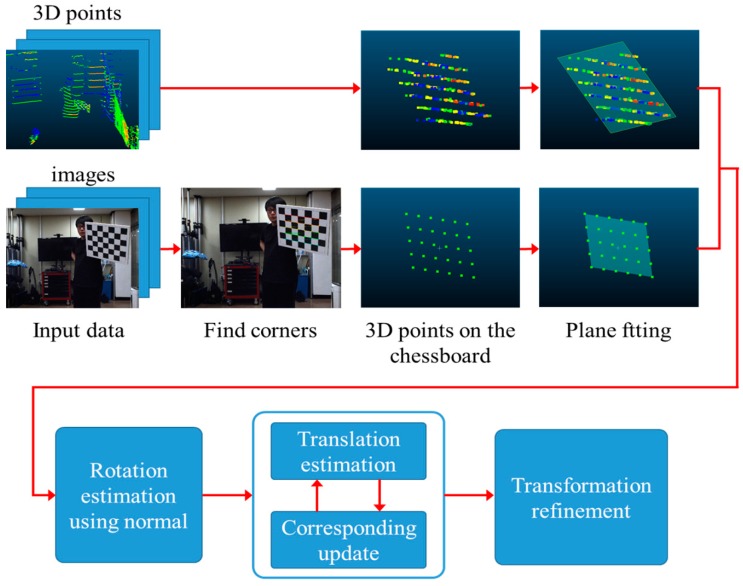
The flowchart of the proposed calibration method. Image and depth data are acquired with many different poses of the chessboard.

In this section, we describe details of the proposed calibration method step by step. A flowchart of the proposed calibration method for a camera–LiDAR sensor unit is shown in Figure 3. A summary of the proposed method is as follows. First, 2D images and 3D distance data of a chessboard are captured from a camera–LiDAR sensor module. We acquire multiple image and distance data at different poses (position and orientation) of the chessboard. At each chessboard pose, the camera pose with respect to the world coordinate system (chessboard coordinate system) is calibrated by the PnP algorithm. Then, the 3D positions of the chessboard corners are calculated by back-projection of the 2D chessboard corners, and they are used to fit to a 3D plane of the chessboard with respect to the camera coordinate system. For the same chessboard pose, the 3D points scanning on the board are segmented by a simple distance filtering. Then, the 3D plane equation with respect to the LiDAR sensor coordinate system is also calculated. The relative orientation between the camera and the LiDAR is estimated by aligning the normal vectors of all plane pairs.

To find the relative translation, we arbitrarily select a 3D point in each rotated camera plane and project the point to another point on the 3D LiDAR plane. The distance between two 3D points in each view is iteratively minimized until all point pairs from all views are properly aligned. Finally, the relative rotation and translation are refined by minimizing the distance between LiDAR planes and all 3D corner points of the chessboard.

### 3.1. 3D Chessboard Fitting Using LiDAR Data

One advantage of the proposed calibration method is using only one common chessboard pattern. Therefore, it is very easy to calibrate the extrinsic parameters between a 2D vision sensor and a 3D LiDAR sensor. In addition, the proposed method can also be used to calibrate multiple cameras and a LiDAR sensor if the camera can capture the chessboard pattern simultaneously.

**Figure 4:**
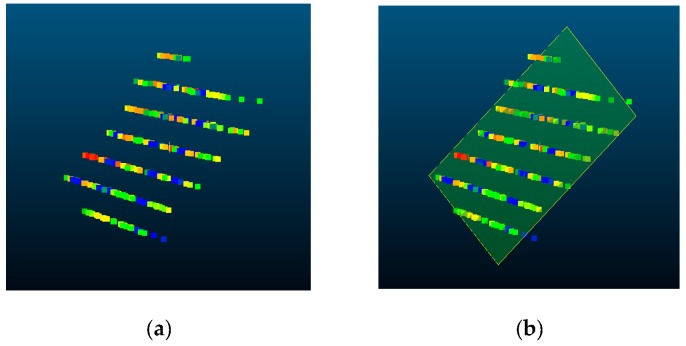
(**a**) 3D LiDAR points in the chessboard area. (**b**) Result of plane fitting.

The first step of calibration is to capture the images and depth data of the chessboard and to find the 3D plane equations of the board in each sensor coordinate system. Suppose the camera and LiDAR sensors simultaneously capture images and 3D depth data of a certain pose of the chessboard. If some portions of the LiDAR scan data contain the depth of the chessboard, it is straightforward to find the 3D plane equation with respect to the LiDAR coordinate system. As shown in Figure 4a, the 3D points only on the chessboard area can be extracted from the LiDAR data by a simple distance filtering. Then, we use the RANSAC [19] algorithm to fit an accurate 3D plane equation. The best-fit plane of 3D points $P_{L_{i}}={\left\{p_{L_{i}}^{1},p_{L_{i}}^{2},\ldots ,\;p_{L_{i}}^{m}\right\}}_{i=\left(1,\ldots ,N\right)}$ of the chessboard at the *i*-th frame is estimated using the inliers determined by the RANSAC algorithm. Here, N is the total number of image frames and m is the number of 3D points in the *i*-th frame. This RANSAC-based plane fitting algorithm can be described as follows:

1. Randomly selecting three points from 3D points $P_{L_{i}}$ on the chessboard.
2. Calculating the plane equation using the selected three points.
3. Finding inliers using the distance between the fitted plane and all the other 3D points.
4. Repeat above steps until finding the best plane with the highest inlier ratio.

In detail, the maximum number of iterations is 1000, and 3D points within 10 mm from the plane are considered as inliers. The plane with an inlier ratio of 90% or more is considered to the best-fit plane.

We can calculate a best-fit plane of the chessboard in the LiDAR coordinate system by employing RANSAC. This plane is used to estimate the relative pose with the camera coordinate system. Figure 4b shows the result of plane fitting using the 3D points of the chessboard.

### 3.2. 3D Chessboard Fitting Using Camera Images

From a 2D image of the camera, we can find the 3D plane equation of the chessboard if we know the intrinsic parameters of the camera. In this paper, we assume that the intrinsic parameters of the camera are calibrated in advance using a common camera calibration technique such as Zhang’s algorithm [20]. In our experiments, we use the intrinsic calibration algorithm implemented in MATLAB. Once we know the intrinsic parameters, the 3D pose of the camera with respect to the chessboard coordinate system is calculated by the well-known PnP algorithm [21] implemented in OpenCV. We cannot use the MATLAB algorithm for camera pose calibration because the algorithm rejects some chessboard images as outliers. This is not allowed for our purpose because we need to use all input pairs of the camera image and LiDAR data.

**Figure 5:**
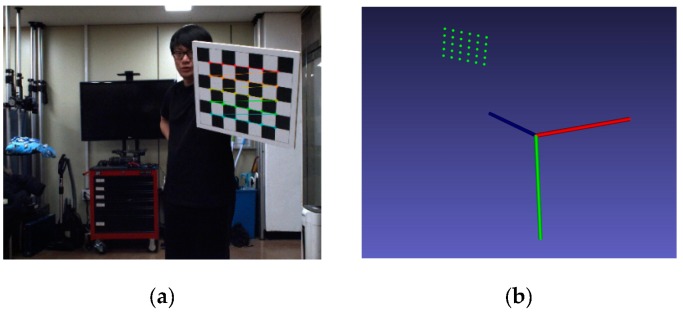
(**a**) Chessboard corners in the camera image are found with subpixel precision. (**b**) All corners are reprojected into 3D space and represented (green) in the camera coordinate system.

Then, the 2D coordinates of all chessboard corners in an image can be reprojected to the 3D coordinates using the chessboard to camera transformation matrix. Because we have all N views of chessboard images, we can find N sets of 3D chessboard corners. Figure 5a shows an image of the chessboard and detected corner points with subpixel precision. These corner points are reprojected into 3D space, as shown Figure 5b, and all the reprojected points are reconstructed with respect to the camera coordinate system.

Finally, 3D planes with respect to the camera coordinate system are fitted using 3D chessboard corners of each view and the PCA (principal component analysis) algorithm. Assuming that $P_{C_{i}}={\left\{p_{C_{i}}^{1},p_{C_{i}}^{2},\ldots ,\;p_{C_{i}}^{m}\right\}}_{i=\left(1,\ldots ,N\right)}$ as a set of 3D chessboard corner points obtained from the *i*-th chessboard pose and $p_{C_{i}}^{j}=\left[x_{C_{i}}^{j},\;y_{C_{i}}^{j},\;z_{C_{i}}^{j}\right]$ is the coordinates of the *j*-th 3D chessboard corner. A rectangle matrix ***A*** composed of elements of $P_{C_{i}}$ is shown in Equation (1) and ***A*** is decomposed to $U\Sigma V^{T}$ using SVD (singular value decomposition). Each element of the fourth row of $V^{T}$ becomes coefficients *a*, *b*, *c*, and *d* of the plane equation ax+by+cz+d=0.

(1) $$A=\left[\begin{array}{c} x_{C_{i}}^{1}\;y_{C_{i}}^{1}\;z_{C_{i}}^{1}\;1\\ x_{C_{i}}^{2}\;y_{C_{i}}^{2}\;z_{C_{i}}^{2}\;1\\ \vdots \\ x_{C_{i}}^{m}\;y_{C_{i}}^{m}\;z_{C_{i}}^{m}\;1 \end{array}\right]$$

### 3.3. Calculating Initial Transformation between Camera and LiDAR Sensors

**Figure 6:**
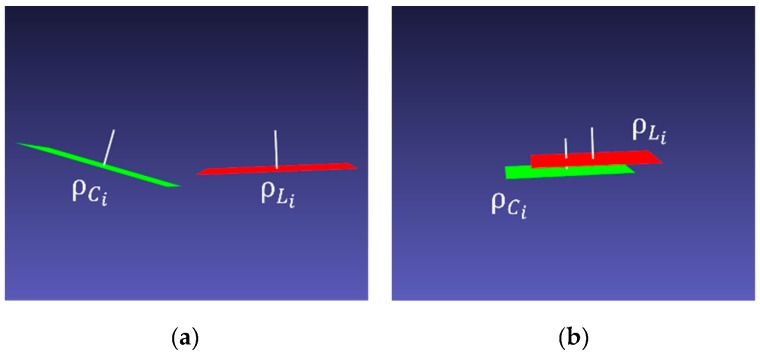
Planes $\rho_{C_{i}}$, $\rho_{L_{i}}$ and normal vectors (white) of the chessboard are fitted by the 3D points of the camera (green) and the LiDAR (red), respectively. (**a**) The initial pose of the two planes and (**b**) the result of applying the calculated rotation matrix.

In the proposed extrinsic calibration, we first estimate the rotation transformation between two sensors, from the camera to the LiDAR coordinate systems. In Figure 6, let $\rho_{C_{i}}$ and $\rho_{L_{i}}$ be the plane fitting results of the *i*-th pose of the chessboard from the camera (*C_i_*) and the LIDAR (*L_i_*) sensors, respectively. The white line starting from the plane represents the normal vector of each plane. The initial poses of two planes are shown in Figure 6a. We consider the LiDAR coordinate system as the world coordinate system. The rotation matrix ***R*** from the camera to the world coordinate systems can be found, which minimizes the error as follows:

(2) $$\varepsilon_{R}=\overset{N}{\underset{i=1}{\sum }}\left|\left(RN_{C_{i}}\right)\cdot N_{L_{i}}-1\right|,$$

where $N_{L_{i}}={\left[n_{L_{i}}^{x},n_{L_{i}}^{y},n_{L_{i}}^{z}\right]}^{T}$ represents the normal vector of the plane $\rho_{L_{i}}$ and $N_{C_{i}}={\left[n_{C_{i}}^{x},n_{C_{i}}^{y},n_{C_{i}}^{z}\right]}^{T}$ represents the normal vector of the plane $\rho_{C_{i}}$. Because the rotation of the normal vector of $\rho_{C_{i}}$ should match with that of

$$\rho_{L_{i}}$$,

we can find the rotation matrix that minimizes the error in Equation (2). The cross-covariance matrix $\Sigma_{N_{C_{i}}N_{L_{i}}}$ of the sets $N_{C_{i}}^{k}$ and $N_{L_{i}}^{k}$ is given by the following:

(3) $$\Sigma_{N_{C_{i}}N_{L_{i}}}=\frac{1}{N}\overset{N}{\underset{i=1}{\sum }}{N_{C_{i}}N_{L_{i}}}^{T}.$$

The matrix $\Sigma_{N_{C_{i}}N_{L_{i}}}$ is decomposed to ${U\Sigma V}^{T}$ using SVD, and the rotation matrix is calculated by Equation (4).

(4) $$R=VU^{T}$$

**Figure 7:**
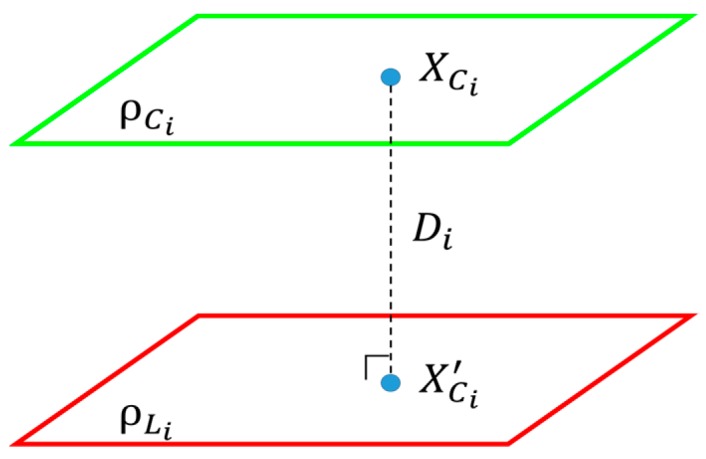
Distance from plane $\rho_{C_{i}}^{\prime }$, to plane $\rho_{L_{i}}$.

If the rotation matrix is calculated correctly, the rotated plane $\rho_{C_{i}}^{\prime }$ must be exactly parallel to $\rho_{L_{i}},$ as shown in Figure 6b. Then, the translation vector is calculated by minimizing the distance between two planes. Figure 7 shows how to estimate the distance between two planes. First, a 3D point $X_{C_{i}}={\left[x_{C_{i}},\;y_{C_{i}},\;z_{C_{i}}\right]}^{T}$ is selected randomly on $\rho_{C_{i}}^{\prime }$. In the actual experiments, we select one of the chessboard corners. The selection is random by considering the imperfect rotation estimation. The translation vector $t={\left[t_{x},t_{y},t_{z}\right]}^{T}$ is calculated by first projecting $X_{C_{i}}$ onto $\rho_{L_{i}}$ and then minimizing the distance between $X_{C_{i}}$ and the projected point $X_{C_{i}}^{\prime }.$ The distance between $X_{C_{i}}^{\prime }$ and $X_{C_{i}}$ in the *i*-th pose can be written as follows:

(5) $$\varepsilon_{t}=\left|\frac{a_{L_{i}}\left(x_{C_{i}}+t_{x}\right)+b_{L_{i}}\left(y_{C_{i}}+t_{y}\right)+c_{L_{i}}\left(z_{C_{i}}+t_{z}\right)+d_{L_{i}}}{\sqrt{a_{L_{i}}^{2}+b_{L_{i}}^{2}+c_{L_{i}}^{2}}}\right|,$$

where the plane equation of $\rho_{L_{i}}$ is given as $a_{L_{i}}x+b_{L_{i}}y+c_{L_{i}}z+d_{L_{i}}=0$.

$X_{C_{i}}^{\prime }$ is calculated using $\varepsilon_{t},$ $X_{C_{i}},$ and the normal vector of the LiDAR plane. If the center of projected points $X_{C_{i}}^{\prime }$ is $\mu_{C_{i}}^{\prime }$ and the center of points $X_{C_{i}}$ on the chessboard plane is $\mu_{C_{i}}$, then the translation shift Δt is calculated by Equation (6). The translation ***t*** is decided by accumulating the shift vector, and the process is repeated until convergence.

(6) $$\Delta t=\mu_{C_{i}}^{\prime }-\mu_{C_{i}}$$

**Figure 8:**
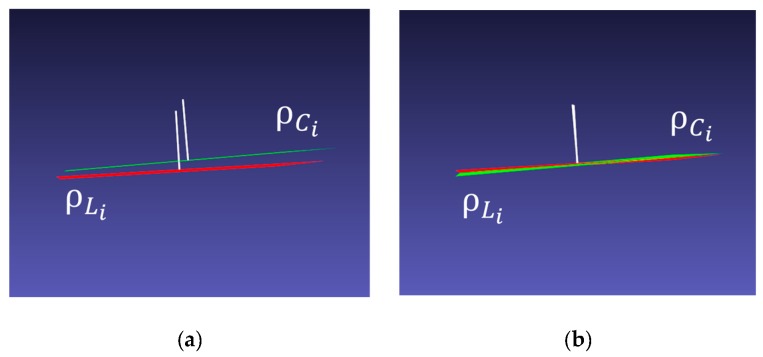
(**a**) The initial position of rotated plane $\rho_{C_{i}}^{\prime }$, and $\rho_{L_{i}}$. (**b**) The result of the planes to which the estimated translation vector is applied.

The translation vector is estimated by minimizing the error in Equation (5) in all poses of the chessboard. Figure 8a shows the initial pose of $\rho_{C_{i}}^{\prime }$ and $\rho_{L_{i}}$ after the initial rotation matrix is applied. Figure 8b shows the alignment between two planes after translation is estimated.

There are three degrees of freedom for rotation and translation. To remove any ambiguity in translation and rotation estimations, at least three poses of the chessboard data are needed. We use three or more frames and a corresponding amount of LiDAR depth data. Therefore, the translation parameters should be solved in an iterative way. In the iteration of the linear optimization method, the sampled 3D point $X_{C_{i}}$ is iteratively projected to plane $\rho_{L_{i}}$ until the distance error is minimized.

### 3.4. Transformation Refinement

More accurate transformation between the camera and LiDAR sensors is calculated based on the initial transformation obtained in Section 3.3. The transformation refinement is performed using the plane equation of LiDAR and all corners of the chessboard. The transformation refinement step is as follows. First, suppose $P_{C_{i}}^{j}$ is the *j*-th corner point of the *i*-th pose of the chessboard, and it is already transformed by the initial transformation. As shown in Equation (7), we define an error function, which is the distance between all corners of the chessboard and the LiDAR planes. Then, the rotation matrix ***R*** and the translation vector ***t*** between the camera and the LiDAR sensors are refined by minimizing the error in Equation (7). We solve the non-linear optimization problem given in Equation (7) using the Levenberg–Marquardt (LM) algorithm.

(7) $$\varepsilon_{T}=\left|\frac{a_{L_{i}}x_{C_{i}}^{j}+b_{L_{i}}y_{C_{i}}^{j}+c_{L_{i}}z_{C_{i}}^{j}+d_{L_{i}}}{\sqrt{a_{L_{i}}^{2}+b_{L_{i}}^{2}+c_{L_{i}}^{2}}}\right|$$

(8) $$\left[\begin{array}{c} x_{C_{i}}^{j}\\ y_{C_{i}}^{j}\\ z_{C_{i}}^{j} \end{array}\right]=RP_{C_{i}}^{j}+t$$

## 4. Experimental Results

### 4.1. Error Analysis Using Simulation Data

**Figure 9:**
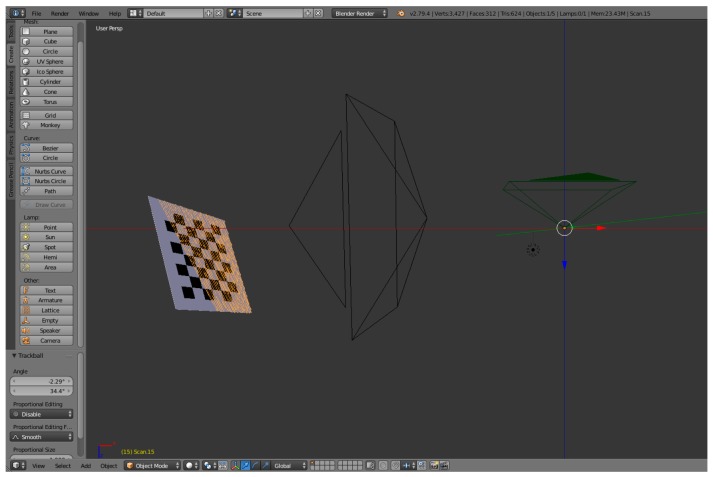
A screen capture of generating a test sequence in Blensor.

As the first experiments, we analyze the accuracy of the proposed algorithm using simulation data and ground truth. We generate test datasets to simulate the 3D LiDAR–camera system using Blensor [22]. The rotation and translation from the camera to the LiDAR are set as [−100°, −5°, 90°] and [−1.2 m, 0.1 m, −0.3 m], respectively. The simulated camera uses an 8.0 mm lens, and the resolution of the captured image is set to 3840 × 2160 pixels. The simulated LiDAR is a 64-channel Velodyne HDL-64E LiDAR sensor (only 32- and 64-ch. are available in Blensor). A sequence of 40 test frames is generated. The test sequence consists of camera images and a corresponding data range of chessboards. In each image and range pair, the chessboard is manually positioned in different poses. Gaussian noise with 0.01 m standard deviation is added to the direction vector of each LiDAR point to simulate the real situation. The maximum noise is set to 0.1 m. Figure 9 shows a screen capture of generating a test sequence in the simulator.

We conducted experiments using the simulation data from minimum of 3 frames to a maximum of 30 frames. The rotation and translation errors are measured with respect to the ground truth by Equations (9) and (10), respectively:

(9) $$E_{R}=\frac{1}{n}\overset{n}{\underset{i=1}{\sum }}\left(\frac{trace\left(I-R_{GT}{R_{e}^{i}}^{T}\right)}{3}\right),$$

(10) $$E_{t}=\frac{1}{n}\overset{n}{\underset{i=1}{\sum }}∥t_{GT}-\;t_{e}^{i}∥.$$

In Equation (9), trace (*X*) is the summation of the diagonal elements of a square matrix *X* and I is the 3 × 3 identity matrix.

In each experiment, test frames are randomly selected from 100 total frames and the experiments is repeated 100 times (*n* = 100). In the above equations, $R_{GT}$ and $t_{GT}$ are the rotation and the translation ground-truth, respectively, between the camera and the LiDAR sensors. $R_{e}$ and $t_{e}$ are estimation results by the proposed algorithm.

**Figure 10:**
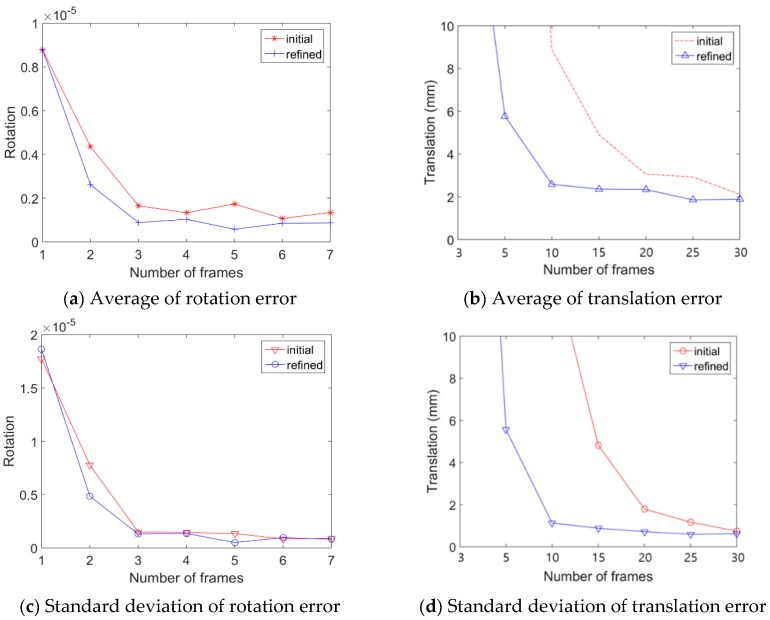
Results of the simulation. (**a**,**b**) Average error between ground truth and the estimated pose, and (**c**,**d**) standard deviation of error.

Figure 10 and Table 1 are the comparison results between ground-truth and estimated extrinsic parameters. We also compared both the initial and the refined extrinsic parameters. The rotation and the translation errors decrease as the number of test frames increases. The refinement results show that calibration error becomes lower compared with the initial error. The mean error of rotation is measured by Equation (9), which measures the absolute difference with the ground truth rotation matrix R_TH_.

### 4.2. Consistency Analysis Using Real Data

We conducted several experiments to evaluate the accuracy of the proposed extrinsic calibration method using the omnidirectional sensing system shown in Figure 1. The LiDAR 3D points and images are captured using PCL (point cloud library) and FLIR SDK on Windows OS. Chessboard corners of images are detected using the OpenCV library. The time synchronization between two sensors is not considered because the LiDAR and camera acquire a static scene. In addition, the time interval between two acquisitions is very short.

To analyze the accuracy of the proposed calibration method, experiments were done with the combination of the following configurations:

- To test in a different field of view, two types of lens are used, that is, a 3.5 mm lens and an 8 mm lens;
- To test with a different number of image frames, a total of 61 and 75 frames are obtained from the 3.5 mm and 8 mm lens, respectively.

There is no ground truth parameter of the external transformation between the camera and the LiDAR sensors. Instead, we measure the following parameters for accuracy and consistency analysis:

- The translation between the coordinate system;
- The rotation angle between the coordinate system along the three coordinate axes;
- The measurement difference between the results of using the 3.5 mm and 8 mm lenses (for consistency check).

**Figure 11:**
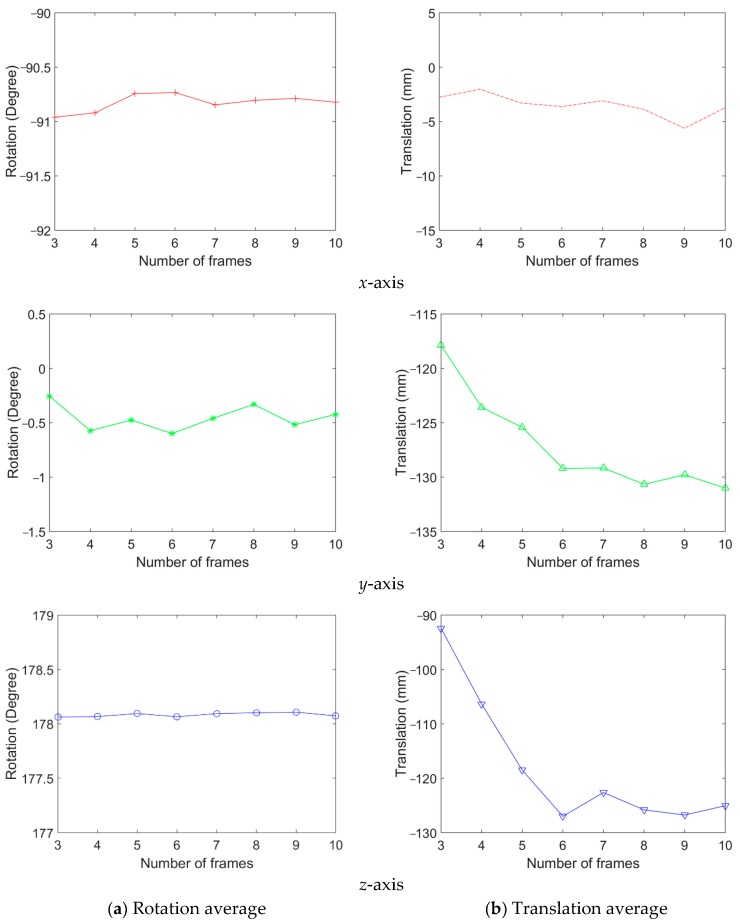
The result of rotation and translation average between the coordinate systems (3.5 mm lens).

**Figure 12:**
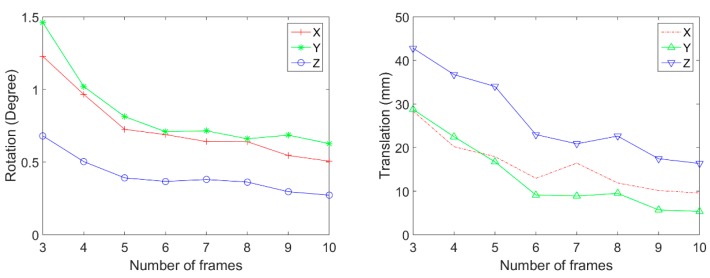
Standard deviation of rotation and translation (3.5 mm lens).

Among all captured image frames of the camera and LiDAR, we randomly select 3 to 10 frames for calibration. We repeated the random selection and the calibration 100 times in each number of image frames. Then, the mean and the standard deviation of calibration results are calculated. Figure 11 shows the rotation and translation average between the coordinate systems. In this result, the 3.5 mm lens is used. This result shows that the average rotation and translation of each axis are stable when six and more frames are used. Figure 12 shows the standard deviation of rotation and translation after 100 repetitions of experiments. The standard deviation converges as the number frames increases.

**Figure 13:**
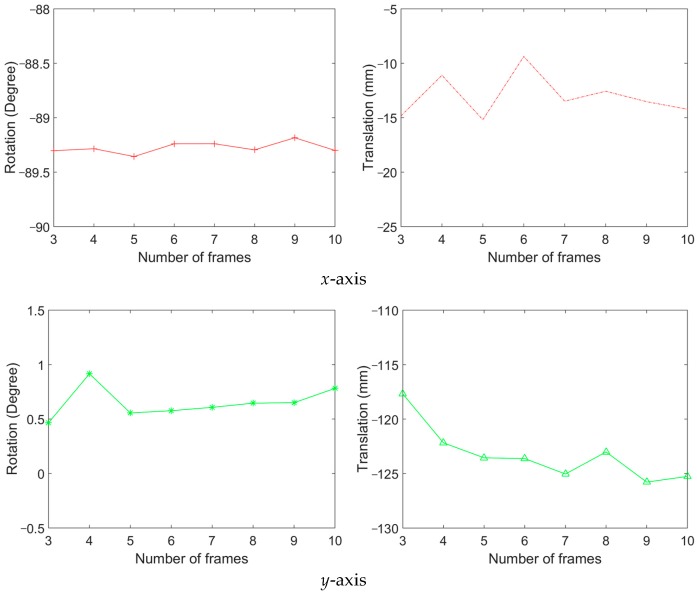

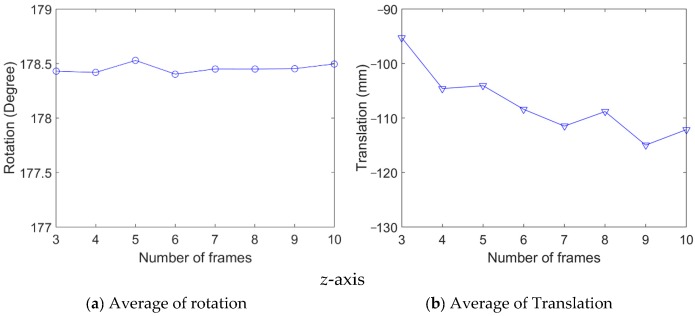
The result of rotation and translation average between the coordinate systems (8 mm lens).

**Figure 14:**
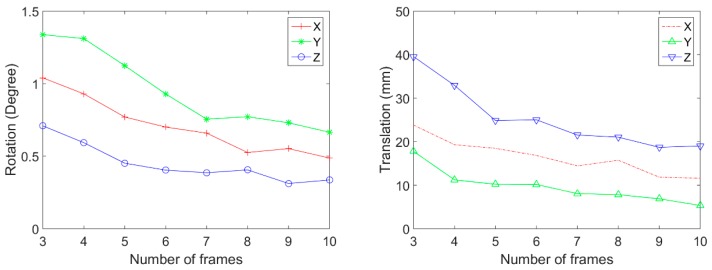
Standard deviation of rotation and translation (8 mm lens).

Figure 13 and Figure 14 shows the average and standard deviation, respectively, of rotation and translation when the 8 mm lens is used. The average rotation does not change after using more than seven frames. The standard deviation also becomes very low after more than seven frames are used. The standard deviation at five frames becomes a little high owing to a random combination of image frames; however, after using more than six frames, the standard deviation decreases as expected.

**Figure 15:**
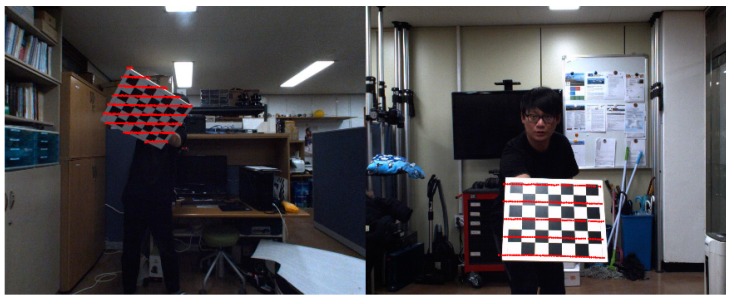

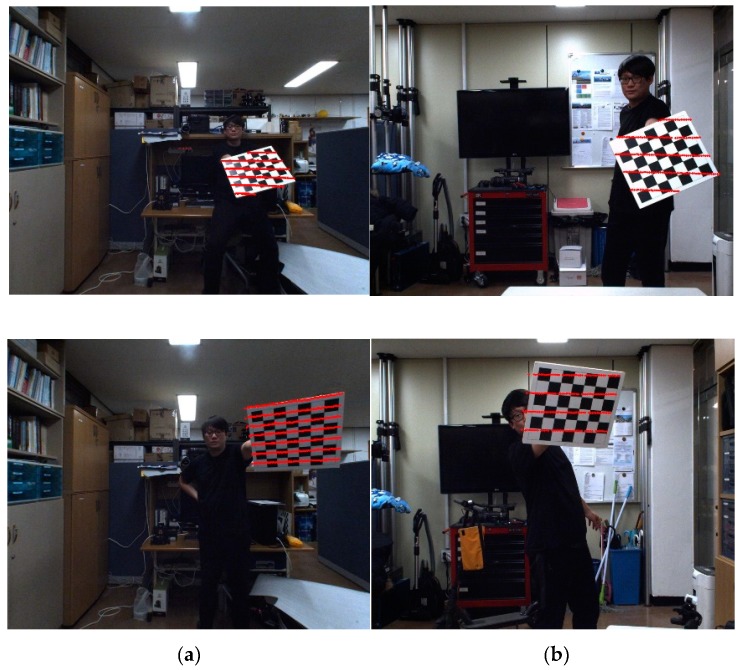
Back-projection examples of 3D points of LiDAR to the images of the camera with (**a**) 3.5 mm and (**b**) 8 mm lenses.

Table 2 shows the result of rotation and translation when using 10 frames. The 10 frames are also randomly selected from the database and this experiment is also done 100 times. In this table, we can see that the average of rotation and translation between the coordinate system is very similar regardless of the focal length of the camera lens. This result shows that the proposed calibration method is accurate and consistent in calibrating the external parameters between the camera and the LiDAR sensors. Table 3 is the result according to the distance between the sensor unit and the chessboard using 10 randomly selected frames. Experiments are performed such that distances between the sensor unit and the chessboard are 2, 3, 4, and 5 m, respectively. The rotation and translation between the sensors in the 2 to 4 m data set have similar averages and low standard deviation regardless of the focal length of the camera lens. However, using the 3.5 mm lens, we cannot detect the chessboard corners in the image captured at a distance of 5 m, and thus there is no result in Table 3. In addition, with the 8 mm lens, the chessboard corner detection at 5 m has low accuracy, which results in inconsistent measurement, as shown in Table 3. The result shows that our algorithm has stable performance from distances of 2 to 4 m between the sensor unit and the chessboard. Using “measure difference” in Table 2 and Table 3, we show how much the results are consistent even with different lens focal lengths. As shown in Table 3, the calibration results are not reliable if the calibration object distance is over 5 m. We propose to use the proposed method using images and LiDAR data that capture chessboards within a distance of 4 m. Figure 15 is the result of the projection of the 3D LiDAR points to the chessboard area in the image planes. The red dots are the projected 3D points of LiDAR that are scanning the chessboard areas.

## 5. Conclusions

In this paper, we propose an extrinsic calibration method to find the 3D transformation between a vision camera and a Velodyne LiDAR sensors. The method is based on matching multiple 3D planesthat are obtained from multiple poses of a single chessboard with respect to the camera and the LiDAR coordinate systems, respectively. The initial rotation is estimated by aligning the normal vectors of the planes, and the initial translation is estimated by projecting a 3D point on a plane in the camera coordinate system to the plane in the LiDAR coordinate system. The initial rotation and translation are then refined using an iterative method. The resulting relative pose information can provide data fusion from both sensors. The fused data can be used to create a 3D map of the environment for navigation of autonomous vehicles.

## Figures and Tables

**Table 1:** The result of a comparison between ground-truth and estimated extrinsic parameters.

	Initial		Refined	
Number of Frames	Rotation (10^−5^) (Standard Deviation)	Translation (mm) (Standard Deviation)	Rotation (10^−5^) (Standard Deviation)	Translation (mm) (Standard Deviation)
3	0.87 (1.77)	133.86 (130.05)	0.87 (1.86)	22.82 (43.13)
5	0.43 (0.77)	38.69 (57.78)	0.26 (0.48)	5.76 (5.56)
10	0.16 (0.15)	8.88 (13.60)	0.08 (0.13)	2.58 (1.12)
15	0.13 (0.14)	4.90 (4.82)	0.10 (0.13)	2.36 (0.87)
20	0.17 (0.13)	3.05 (1.79)	0.05 (0.05)	2.34 (0.71)
25	0.10 (0.08)	2.92 (1.16)	0.08 (0.09)	1.85 (0.59)
30	0.13 (0.09)	2.11 (0.74)	0.08 (0.08)	1.88 (0.61)

**Table 2:** The result of rotation and translation after 10 iterations.

Focal Length	Rotation (Degree) (Standard Deviation)			Translation (mm) (Standard Deviation)		
	X	Y	Z	X	Y	Z
3.5 mm	−89.300 (0.487)	−0.885 (0.517)	178.496 (0.335)	−14.22 (11.60)	−125.26 (5.34)	−112.13 (19.00)
8.0 mm	−90.822 (0.506)	−0.598 (0.460)	178.073 (0.272)	−3.72 (9.56)	−131.00 (5.34)	−125.05 (16.36)
Measure difference	1.522	−0.287	0.423	10.49	−5.74	−12.91

**Table 3:** The result according to the distance between the sensor unit and the chessboard.

Focal Length	Distance (m)	Rotation (Degree) (Standard Deviation)			Translation (mm) (Standard Deviation)		
		X	Y	Z	X	Y	Z
3.5 mm	2	−88.896 (0.569)	1.131 (1.434)	177.934 (0.351)	−17.46 (14.43)	−129.92 (5.20)	−107.90 (26.21)
	3	−88.960 (0.746)	0.337 (2.022)	178.359 (0.538)	−6.05 (19.77)	−123.94 (8.39)	−89.90 (50.69)
	4	−89.6116 (1.170)	0.106 (2.294)	178.693 (0.788)	−35.89 (56.83)	−125.08 (17.08)	−92.04 (76.45)
	5	−	−	−	−	−	−
8.0 mm	2	−88.624 (0.485)	−0.236 (0.653)	177.981 (0.349)	−3.80 (15.65)	−147.55 (3.29)	−83.52 (15.66)
	3	−87.766 (0.570)	0.249 (0.899)	177.984 (0.461)	3.56 (22.07)	−145.37 (5.78)	−123.69 (28.05)
	4	−88.033 (1.009)	−0.100 (1.744)	178.533 (0.483)	−36.87 (38.57)	−133.52 (8.18)	−133.52 (66.78)
	5	−85.222 (4.115)	3.345 (7.675)	178.222 (1.214)	−58.58 (93.93)	−114.38 (24.91)	−310.56 (319.27)
Measure difference	2	−0.271	1.368	−0.046	−13.65696	17.62756	24.37621
	3	−1.193	0.087	0.375	−9.61441	21.42924	−32.97788
	4	−1.578	0.207	0.159	0.98018	8.43786	−37.84989
	5	−	−	−	−	−	−
